# Tissue poromechanical deformation effects on steam pop likelihood in 3-D radiofrequency cardiac ablation

**DOI:** 10.1186/s13036-023-00365-5

**Published:** 2023-08-07

**Authors:** Patcharaporn Wongchadakul, Ashim K. Datta, Phadungsak Rattanadecho

**Affiliations:** 1 Princess Srisavangavadhana College of Medicine, Chulabhorn Royal Academy, Bangkok, Thailand; 2 Department of Biological & Environmental Engineering, Cornell University, Ithaca, NY USA; 3 Center of Excellence in Electromagnetic Energy Utilization in Engineering (C.E.E.E.), Department of Mechanical Engineering, Faculty of Engineering, Thammasat University, Pathumthani, Thailand

**Keywords:** Radiofrequency cardiac ablation, Finite element, Porous media, Poromechanical, Deformation, Steam pop

## Abstract

Radiofrequency Cardiac Ablation (RFCA) is a common procedure that heats cardiac tissue to destroy abnormal signal pathways to eliminate arrhythmias. The complex multiphysics phenomena during this procedure need to be better understood to improve both procedure and device design. A deformable poromechanical model of cardiac tissue was developed that coupled joule heating from the electrode, heat transfer, and blood flow from normal perfusion and thermally driven natural convection, which mimics the real tissue structure more closely and provides more realistic results compared to previous models. The expansion of tissue from temperature rise reduces blood velocity, leading to increased tissue temperature, thus affecting steam pop occurrence. Detailed temperature velocity, and thermal expansion of the tissue provided a comprehensive picture of the process. Poromechanical expansion of the tissue from temperature rise reduces blood velocity, increasing tissue temperature. Tissue properties influence temperatures, with lower porosity increasing the temperatures slightly, due to lower velocities. Deeper electrode insertion raises temperature due to increased current flow. The results demonstrate that a 5% increase in porosity leads to a considerable 10% increase in maximum tissue temperature. These insights should greatly help in avoiding undesirable heating effects that can lead to steam pop and in designing improved electrodes.

## Introduction

Radiofrequency Cardiac Ablation (RFCA) is a common procedure to treat arrythmia, a symptom of abnormal heart rhythm. RFCA works by electrically heating cardiac tissue to destroy abnormal signal pathways. In RFCA, a radiofrequency electrode catheter is pressed against the cardiac tissue allowing current to flow through the tissue to the ground in another location of the tissue [1]. Complications in this procedure have been reported that include steam pop and char [2, 3] due to uncontrolled high temperatures in regions. Steam pop from evaporation represents a potentially severe complication of RFCA, which is associated with embolic stroke, cardiac perforation, and ventricular septal defect. Thus, preventing steam pop is clinically desirable [4]. The temperatures in RFCA are the result of complex physical processes involving electrical heating, thermal conduction and convection, blood flow, and tissue deformation that depend on several device (e.g., electrode design and size) and procedure (electrode insertion depth, power level, duration of heating, nature of the tissue) factors.

Initial mechanistic modeling of RFCA [5–8] included modeling of voltage equation and heat conductive equation. Fluid (blood) flow in the tissue was not included in these studies, except through blood flow-contributed heat source term in the bioheat equation. Fluid (blood) flow over the tissue in the cardiac chamber and the resulting convective heat transfer between the blood and the tissue is also important and these were included in subsequent studies [9–14]. Eventually, porous media approach to tissue with blood flow [15–20] through it was considered an improvement over the bioheat model [21] and was used for RFCA [22]. In porous media models, tissue is categorized as a vascular region (blood phase) and an extra-vascular region (solid matrix) [23, 24] with the blood flow inside vessels embedded in tissue replaced by an equivalent porosity of the tissue with blood flow through it. However, in these porous media models [22], thermally-driven natural convection and deformation effects were not included.

Comprehensive mechanistic understanding of the electro-thermo-fluid-deformation processes will help improve procedures and design electrodes that reduce complications from overheating. A porous medium approach, used in several bio-heat and mass transfer studies [15–20] has the potential to greatly improve our understanding of the heating process. To have a more realistic picture of the RFCA process, it is important to explain the effect of tissue elasticity and the presence of natural convection. While elasticity was considered [25], fluid flow through the tissue was not included.

Several additional factors affect the heating process. During the systole (tissue contraction) and the diastole (tissue expansion) phases of the cardiac cycle, the depth of inserted electrodes can be different due to tissue deformation [26]. This changes the contact area, changing the current flow and heat generation, an effect that has been studied [26–28]. However, these models did not include the multiphase (solid–liquid) porous media approach with shortcomings as discussed above. Porosity and permeability have been shown to affect thermal transport in other porous media studies [29]. Vessel diameter effect can be included through changing porosity and permeability [22] that affect blood perfusion and thus temperatures relevant to steam pop in RFCA. Porosity and permeability effects, therefore, should be studied in this heat transfer process.

### Specific objectives

Our objectives are to develop a comprehensive model of the RFCA that treats the tissue as a deformable porous medium with fluid (blood) flow through it from the normal perfusion together with thermally driven natural convection as temperature gradients are developed in the tissue. Temperature gradients from electrical heating drive the thermomechanical deformation of the tissue. This combination of physics to make the model more realistic is being presented for the first time. Our objectives are to relate the likelihood of steam pop to: 1) transient and spatial temperature and velocity profiles during the RFCA as affected by poromechanical deformation of the tissue driven by thermal gradients, 2) tissue properties, and 3) effect of electrode insertion depth and the resulting temperature pattern.

### Overview of this manuscript

An electro-thermo-fluid-poromechanical model is developed starting with the computational domains of electrode, deformable tissue with blood flow through it, and blood flow over the tissue. A quasistatic electric field and heat generation from it is modeled using the voltage equation. Fluid flow is set up as Navier–Stokes equations in the blood chamber and, in the tissue, as Darcy-Brinkman equations for flow through porous media. Energy equation is set up with RF and metabolic heat generation. Thermomechanical deformation equations for the tissue are used from the solid mechanics, with temperature gradient as the driving force. The coupled equations are solved numerically and validated against available experimental data. Results are presented in terms of transient temperatures, velocities, and steam pop likelihood, as affected by poromechanical deformation, inserted electrode depth and tissue properties.

## Problem formulation

The key factor in the RFCA procedure's success is controlling the volume of the elevated temperature to achieve effective thermal necrosis of the target tissue, while avoiding an undesired incident due to excessive temperature rise and possible steam pops [1, 2, 30, 31]. Normal body temperature is 37 °C. The myocyte cell death occurs when heated to 50–56 °C for 60 s [32], and instantaneously to 60 °C or higher [33]. Between 60 °C and 100 °C, the mechanisms involved in cell death include desiccation (process of drying) and protein coagulation. When the temperature reaches close to 100 °C, cellular vaporization occurs, resulting in pressure development that can lead to steam pop [34].

**Fig. 1:**
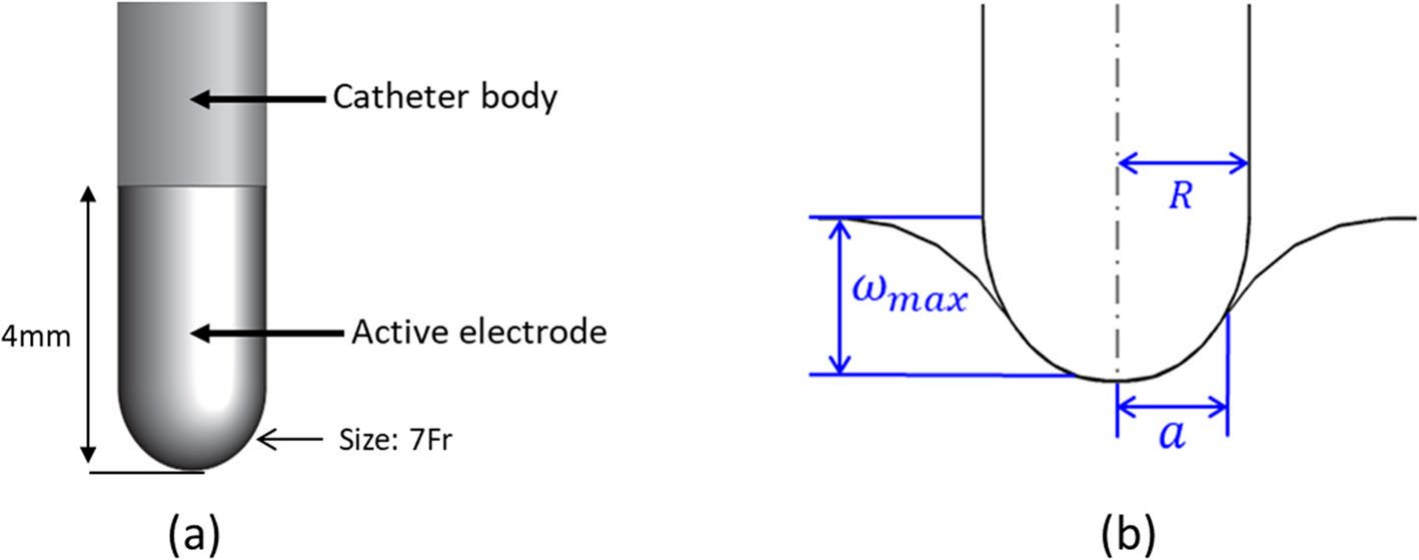
**a** Model geometry of close-irrigated ablation catheter, **b** Cross-section of contact between the electrode tip and cardiac tissue (dimensions data from manufacturer: tip electrode length is 4 mm, tip electrode size is 7 Fr (French scale or French gauge system, commonly used to measure the size of a catheter), and tip electrode diameter (= 2a) is 2.33 mm [35–39])

An electro-thermo-fluid-poromechanical model has been formulated to predict the current flow, resulting temperature rise as affected by fluid flow that includes normal perfusion as well as buoyant flow from temperature gradients, and thermomechanical deformation (expansion) due to temperature rise, all physics coupled together. The geometries of the ablation catheters are indicated in Fig. 1a. The elasticity of cardiac tissue, which deform from inserted electrode, is taken into account and can be evaluated from Eq. 1, illustrated in Fig. 1b. Two different electrode insertion depths are considered in this study, for which different model geometries are created.

In medical literature, steam pops are known complications in ablation procedures that occur when tissue temperatures approach 100 °C [4]. This study aims to address steam pop possibilities by incorporating the water-to-vapor phase change in the thermal model and examining the factors influencing higher temperatures. Recognizing the importance of steam pops, even the smallest temperature variation can have significant physiological and physical implications [40]. In this study, the likelihood of steam pops (indirectly through higher temperatures) is simulated using the heat equation with its conduction and flow terms, along with electrical heat generation and the enthalpy method for the water-to-vapor transition.

### Geometry

During the ablation procedure, an electrode is pushed into the cardiac tissue, resulting in tissue deformation. Electrode radius and the depth to which it is pushed into the tissue affects the contact surface area between the electrode tip and tissue, affecting current flow and temperature rise. Thus, deformation of the cardiac tissue should be considered. Two geometries are created in this study with inserted electrode depths of 1 mm and 3 mm, respectively. Contact radius between electrode and tissue (Fig. 1) can be calculated from geometric considerations as [26, 27]

1 $${\omega }_{max}=\frac{a}{2}\mathrm{log}\left(\genfrac{}{}{0pt}{}{R+a}{R-a}\right)$$

where ${\omega }_{max}$ is maximum vertical displacement (mm), *a* is contact radius (mm) and *R* is the radius of the electrode tip (mm).

## Problem formulation and computational methodology

**Fig. 2:**
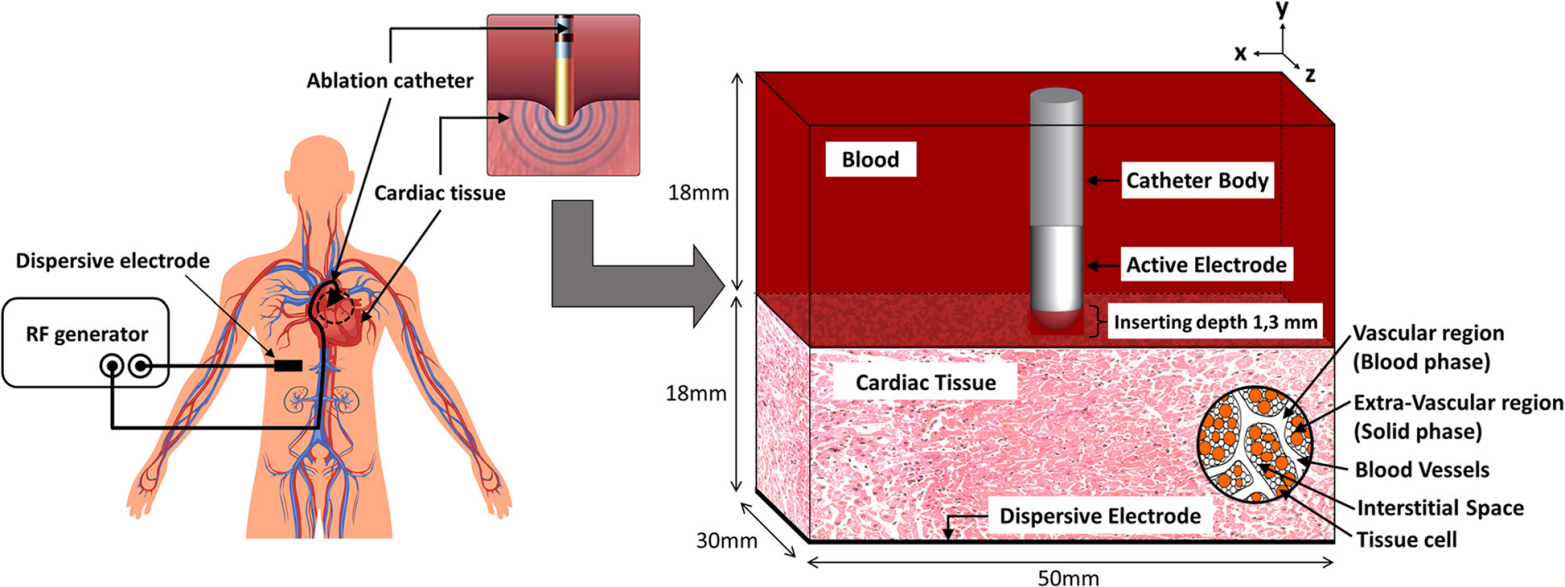
A schematic of the 3-D Radiofrequency Cardiac Ablation (RFCA) procedure and the computational domain

**Fig. 3:**
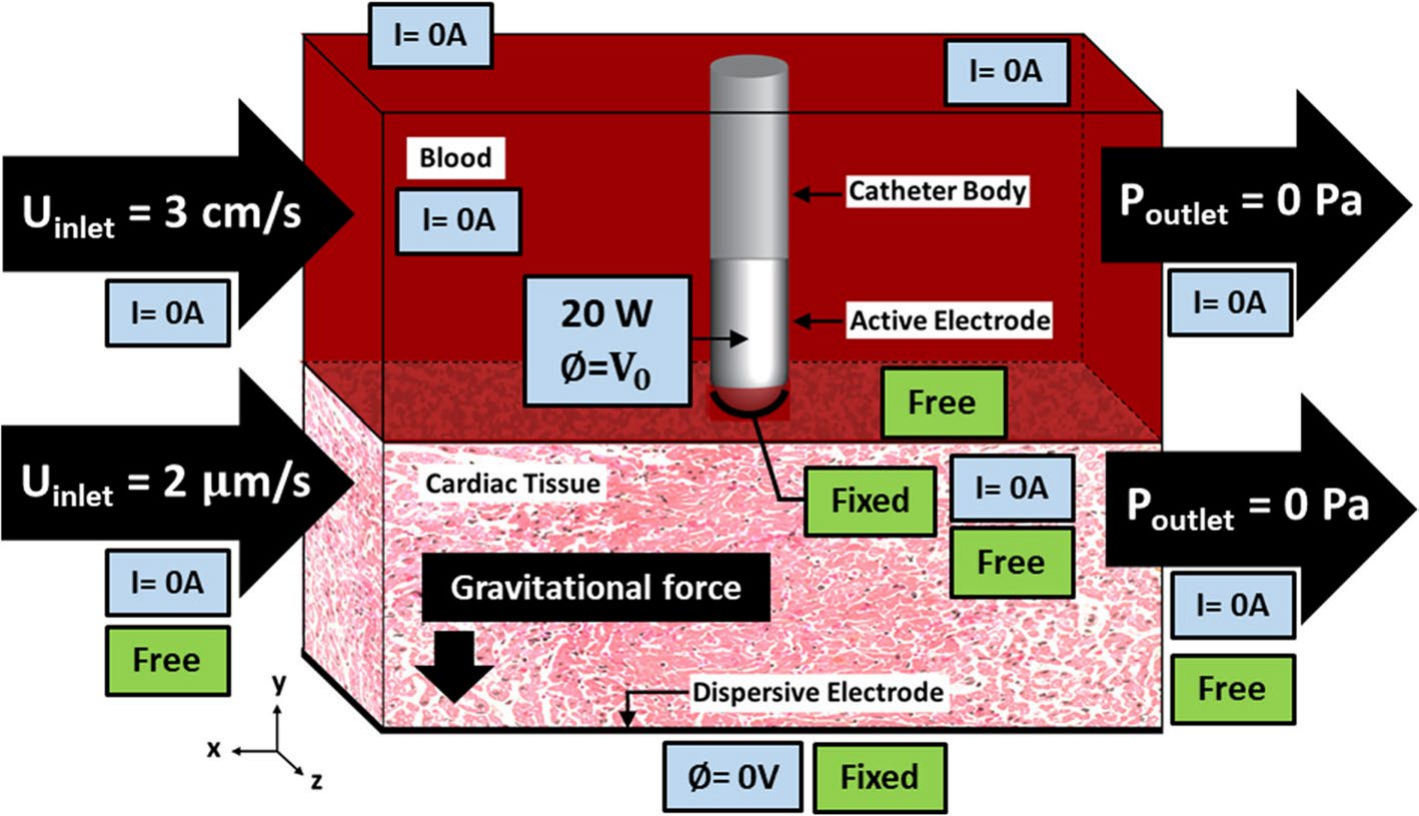
A detailed schematic of the RFCA computational model geometry and boundary conditions used for electrical, thermal, fluid flow, and deformation analysis

Figure 2 shows an overview of the RFCA for arrhythmias, including blood in the cardiac chamber and the porous cardiac tissue with the inserted (pushed) radiofrequency ablation catheter. The ablative catheter consists of the catheter body and the active electrode made from polyethylene (PE) and platinum-iridium alloy (Pt-Ir). Figure 3 shows the 3-D computational schematic with domains and boundary conditions. Table 1 lists the model thermal, electrical and mechanical properties, along with other parameters. The mechanistic model with a porous media approach for RFCA, combined with an analysis of electrical, thermal, poromechanical deformation, and fluid transport, is simulated to obtain the results in this study.

**Table 1:** Input parameters including electrical, thermophysical, mechanical, and other properties used in numerical computations of the RFCA model

**Element/Material**	**Electrode**	**Catheter**	**Cardiac tissue**	**Blood**	**Reference**
	**Pt-Ir**	**PE**			
$\sigma$ (S m^−1^)	4.6 × 10^6^	10^–5^	${\sigma }^{*}$	0.667	[7, 8, 10–14, 22, 25]
*k* (W m^−1^ K^−1^)	71	0.026	${k}^{*}$	0.541	
*p* (kg m^−3^)	21.5 × 10^3^	70	Liquid phase; 1060 Gas phase; 370	1000	
C*p* (J kg^−1^ K^−1^)	132	1045	Liquid phase; 1060 Gas phase; 370	4810	
$\varepsilon_r$ (at 500 kHz)	1.0003	2.26	8 × 10^4^	4 × 10^3^	[41–44]
$\mu$ (kg m^−1^ s^−1^)	-	-	-	0.0035	[26, 27, 45, 46]
$\beta,\;\alpha$ (C° − 1)	-	-	1.23 × 10^–4^	4.5 × 10^–4^	[46, 47]
*v* (-)	-	-	0.499	-	[26–28]
*E* (Pa)	-	-	0.5 × 10^6^	-	[48–50]
Porous media					
* K*(m^2^)	-	-	Blood vessel size of 30 μm; 3.5166 × 10^–11^ Blood vessel size of 5 μm; 1.3823 × 10^–15^	-	-
$\varepsilon$ (-)	-	-	Blood vessel size of 30$\mu$m; 0.1875 Blood vessel size of 5$\mu$m; 0.0313	-	

List of assumptions made to simplify the model include: 1) there is no chemical reaction in the tissue, 2) the steam pop is described by tissue vaporization effect [9, 10, 13, 22, 51], with temperatures above 99 °C, 3) The contact surface between the blood and the tissue is smooth for flow modeling, 4) cardiac tissue is a porous medium saturated with blood, 5) deformation of porous tissue occurs due to temperature gradient, 6) local thermal equilibrium exists between the tissue and the blood flowing through it, leading to both the tissue and the blood being at one temperature at a location, 7) permeability and porosity values are isotropic and for non-infarcted tissue [22, 52], 8) blood convection occurs at the interface of blood and tissue, 9) convective heat transfer between the blood and the cardiac tissue can be described by a heat transfer coefficient, 10) initial tissue temperature is 37℃, the normal body temperature, and 11) electrical conductivity and thermal conductivity values are functions of temperature.

### Electrical analysis

For radiofrequency heating near 500 kHz in RFCA, a quasistatic electric field approximation can be used since the wavelength in the tissue far exceeds tissue thickness, leading to primarily resistive heating. The rate of heat generation, ${Q}_{RF}$ is given by

2 $${Q}_{RF}=\sigma {\left|\mathrm{\rm E}\right|}^{2}$$

where $\left|\mathrm{\rm E}\right|$ is the magnitude of the electric field (V m^−1^) and $\sigma$ is the electrical conductivity of the tissue at the particular frequency (S m^−1^). The electric field can be calculated from the gradient of the voltage:

3 $$\mathrm{\rm E}=-\nabla \varphi$$

where $\varphi$ is electric potential or voltage (V). The governing equations for electric potential are given as Eq. (4) [5, 22, 53].

4 $$\nabla \cdot (\sigma \nabla \varphi )=0$$

#### Boundary condition for voltage equation

**Fig. 4:**
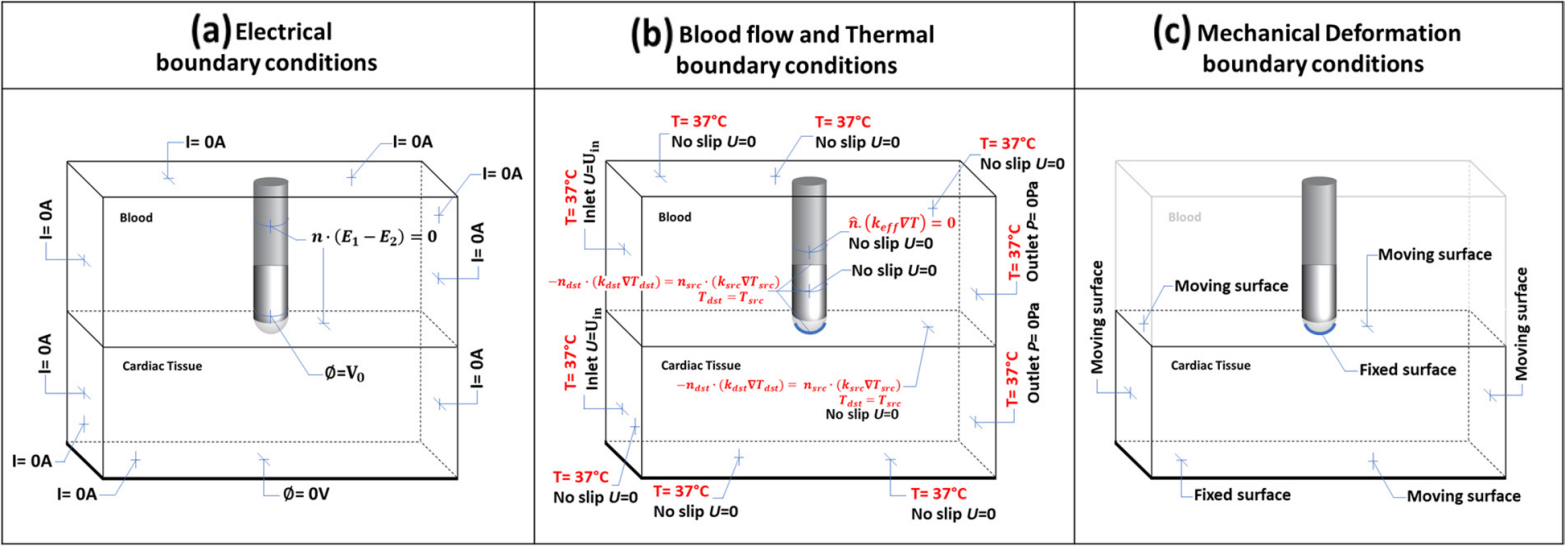
Detailed boundary conditions for **a** voltage, **b** blood flow and thermal, and **c** poromechanical deformation

Figure 4a shows the boundary conditions for electrical analysis. A constant radiofrequency power is used. The input power is set at 20W, for which the voltage used is 25 V. A voltage boundary condition is applied at the active electrode surface, while a zero flux of the electric field is imposed on all other surfaces of the catheter body (at these surfaces, catheter is electrically insulated from the blood or the cardiac tissue).

5 $$n\cdot \left({E}_{1}-{E}_{2}\right)=0$$

The voltage on the bottom surface is set to 0 V to model as a dispersive electrode.

### Blood flow and heat transfer analysis

Governing equations for blood velocity and temperature profiles in the cardiac tissue treats the tissue as a porous medium. The transient momentum equations (Brinkman extended Darcy model) and transient energy equations are used for analyzation [54].

#### Heat conduction equation

Conduction heat transfer in the catheter and electrode is given by:

6 $${\left(\frac{{\partial }^{2}T}{\partial {x}^{2}}+\frac{{\partial }^{2}T}{\partial {y}^{2}}+\frac{{\partial }^{2}T}{\partial {z}^{2}}\right)}_{c,e}=\frac{1}{{\alpha }_{c,e}}\frac{\partial {T}_{c,e}}{\partial t}$$

where $\alpha$ is the thermal diffusivity of the catheter or the electrode.

#### Momentum equations

Blood flow in the blood chamber is described by the continuity equation (Eq. 7) and the Navier–Stokes equations (Eq. (8)).

7 $$\frac{\partial u}{\partial x}+\frac{\partial v}{\partial y}+\frac{\partial w}{\partial z}=0$$

8 $$\begin{array}{l}\rho_b\left(\frac{\partial u}{\partial t}+u\frac{\partial u}{\partial x}+v\frac{\partial u}{\partial y}+w\frac{\partial u}{\partial z}\right)=-\frac{\partial p}{\partial x}+\mu\left(\frac{\partial^2u}{\partial x^2}+\frac{\partial^2u}{\partial y^2}+\frac{\partial^2u}{\partial z^2}\right)\\\rho_b\left(\frac{\partial v}{\partial t}+u\frac{\partial v}{\partial x}+v\frac{\partial v}{\partial y}+w\frac{\partial v}{\partial z}\right)=-\frac{\partial p}{\partial y}+\mu\left(\frac{\partial^2v}{\partial x^2}+\frac{\partial^2v}{\partial y^2}+\frac{\partial^2v}{\partial z^2}\right)-\rho_bg_y\alpha_b\left(T-T_b\right)\\\rho_b\left(\frac{\partial w}{\partial t}+u\frac{\partial w}{\partial x}+v\frac{\partial w}{\partial y}+w\frac{\partial w}{\partial z}\right)=-\frac{\partial p}{\partial z}+\mu\left(\frac{\partial^2w}{\partial x^2}+\frac{\partial^2w}{\partial y^2}+\frac{\partial^2w}{\partial z^2}\right)\end{array}$$

Blood flow in porous tissue is described by the Brinkman extended Darcy equation (Eq. (9)) [55] that has also been used for muscles near an arteries [21]. The buoyancy term is included to account for thermally driven natural convection of blood flow:

9 $$\begin{array}{l}{\rho }_{b}\left(\frac{\partial u}{\partial t}+u\frac{\partial u}{\partial x}+v\frac{\partial u}{\partial y}+w\frac{\partial u}{\partial z}\right)=-\frac{\partial p}{\partial x}+\frac{\mu }{\varepsilon }\left(\frac{{\partial }^{2}u}{\partial {x}^{2}}+\frac{{\partial }^{2}u}{\partial {y}^{2}}+\frac{{\partial }^{2}u}{\partial {z}^{2}}\right)-\frac{\mu }{K}u\\ {\rho }_{b}\left(\frac{\partial v}{\partial t}+u\frac{\partial v}{\partial x}+v\frac{\partial v}{\partial y}+w\frac{\partial v}{\partial z}\right)=-\frac{\partial p}{\partial y}+\frac{\mu }{\varepsilon }\left(\frac{{\partial }^{2}v}{\partial {x}^{2}}+\frac{{\partial }^{2}v}{\partial {y}^{2}}+\frac{{\partial }^{2}v}{\partial {z}^{2}}\right)-\frac{\mu }{K}v-{\rho }_{b}{g}_{y}{\alpha }_{b}\left(T-{T}_{b}\right)\\ {\rho }_{b}\left(\frac{\partial w}{\partial t}+u\frac{\partial w}{\partial x}+v\frac{\partial w}{\partial y}+w\frac{\partial w}{\partial z}\right)=-\frac{\partial p}{\partial z}+\frac{\mu }{\varepsilon }\left(\frac{{\partial }^{2}w}{\partial {x}^{2}}+\frac{{\partial }^{2}w}{\partial {y}^{2}}+\frac{{\partial }^{2}w}{\partial {z}^{2}}\right)-\frac{\mu }{K}w\end{array}$$

where *u*, *v,* and *w* are the blood velocity components (m s^−1^), subscript b represents the blood phase, $\rho$ is density (kg m^−3^), *p* is the pressure (Pa), $\mu$ is kinematic viscosity of blood (kg m^−1^ s^−1^), $\varepsilon$ is the tissue porosity, which is the ratio of the volume fraction of the vascular space, *K* is the permeability (m^2^), ${\beta }_{t}$ is thermal expansion coefficient (${\mathrm{^\circ{\rm C} }}^{-1}$), and $g$ is gravity (m s^−2^). The flow equations for the blood layer are derived from Eq. 9 as a special case of $\varepsilon =1 \mathrm{\ and} \ K=\infty$, when they revert to the Navier–Stokes equations.

The hydraulic permeability of the matrix is estimated as 3.5166 × 10^–11^ m^2^, using the Eq. (20):

10 $$K=\frac{1}{8\tau }\sum \Delta \kappa {r}^{2}$$

where *K* is the permeability (m^2^), $\tau$ is the tortuosity ($\tau =\sqrt{\varepsilon })$, $\Delta \kappa$ is the volume fraction of pores, $\varepsilon$ is the tissue porosity, and *r* is the radius of pores or vessels within tissue ($\mu$m). The capillary diameter, *d*, of cardiac tissue is estimated as 30 $\mu$m [56]. A tissue porosity of 0.1875 is used, which is estimated using the hydraulic diameter Eq. [22, 57, 58]:

11 $$d=\frac{4\varepsilon }{{S}_{v}}$$

where *S*_*v*_ is a specific surface area (25,000 m^−1^) [22, 59]. In this study, tissue porosity is due to the presence of the blood vessels whose sizes (30 m$\mu$ and 5 m$\mu$) are used to compute porosity and permeability, which are shown in Table 1.

#### Energy equations

Local thermal equilibrium can serve as a good approximation for the temperature field for certain applications involving blood vessels of small sizes [21]. The radiofrequency heat generation, as well as metabolic heat, are considered. The energy equation for the blood layer is given by:

12 $${\left(\rho {c}_{p}\right)}_{b}\frac{\partial {T}_{b}}{\partial t}+{\left(\rho {c}_{p}\right)}_{b}{\left(u\frac{\partial T}{\partial x}+v\frac{\partial T}{\partial y}+w\frac{\partial T}{\partial z}\right)}_{b}={k}_{b}{\left(\frac{{\partial }^{2}T}{\partial {x}^{2}}+\frac{{\partial }^{2}T}{\partial {y}^{2}}+\frac{{\partial }^{2}T}{\partial {z}^{2}}\right)}_{b}+{Q}_{RF}$$

while energy equations for porous cardiac tissue layer are given by:

13 $${\left(\rho {c}_{p}\right)}_{eff}\frac{\partial T}{\partial t}+{\left(\rho {c}_{p}\right)}_{eff}\left(u\frac{\partial T}{\partial x}+v\frac{\partial T}{\partial y}+w\frac{\partial T}{\partial z}\right)={k}_{eff}\left(\frac{{\partial }^{2}T}{\partial {x}^{2}}+\frac{{\partial }^{2}T}{\partial {y}^{2}}+\frac{{\partial }^{2}T}{\partial {z}^{2}}\right)+{Q}_{met}+{Q}_{RF}$$

14 $${\left(\rho {c}_{p}\right)}_{eff}=\left(1- \varepsilon \right){\left(\rho {c}_{p}\right)}_{s}+ \varepsilon {\left(\rho {c}_{p}\right)}_{b}\mathrm{\ and} {\ k}_{eff}=\left(1- \varepsilon \right){k}_{s}+ \varepsilon {k}_{b}$$

In these equations, *T* is the temperature, ${c}_{p}$ is the heat capacity, $k$ is the thermal conductivity. Subscripts *eff*, *s* and _*b*_ represent the effective value, solid, and blood phases, respectively, and *u, v* and *w* are the velocities in x, y, and z directions, respectively. The metabolic heat generation rate ${Q}_{met}$, is 684 W m^−3^ [60], which is the basal metabolic rate at a muscle in the thorax, that is the number of calories the body needs to accomplish its most basic life-sustaining functions. The radiofrequency heat source, ${Q}_{RF}$, is equal to the resistive heat generated by the electric field (Eq. (2)).

#### Boundary condition for blood flow and heat transfer

As shown in Fig. 6b, the boundary temperatures of the porous cardiac tissue domain as well as the blood, domain are fixed at 37 °C. The outer surface between the catheter and the blood domain is considered an adiabatic boundary condition:

15 $$\widehat{n}.\left({k}_{eff}\nabla T\right)=0$$

In the blood domain, the inlet velocity of blood in the x-direction, *U*_*inlet*_, is 3 cm s^−1^ [61], and the outlet pressure is set to zero. For the porous tissue domain, *U*_*inle*t_ is assumed to be 2 m$\upmu$ s^−1^ and 0.72 m$\upmu$ s^−1^ corresponding to the same blood flow rate within capillary diameters of 30 m$\upmu$ [56] and 5 m$\mu$ [62], respectively. The outlet pressure is set to zero, as for the blood domain. At blood-catheter and blood-electrode interfaces and the remaining boundaries of the blood and porous cardiac tissue domains, no-slip conditions are applied.

#### Thermophysical properties

When temperature reaches 100 °C, water boils and tissue vaporizes with production of a steam pop incident [4]. The evaporation effects should be taken into consideration in this study. In medical terms, the incidence of steam pop may produce complications after the ablation procedure. Therefore, it should be prevented for effectiveness of ablation surgery outcome. The enthalpy method phase change related to temperature [9, 10, 13, 22, 51] is used for analysis, as given in Eq. (16). The density and heat capacity of both phases (liquid and gaseous) of cardiac tissue are denoted in Table 1.

16 $$\left\{\rho {c}_{p}\right\}_{\begin{array}{c}blood\,within\\ porous\,tissue\end{array}} = \left\{\begin{array}{ll} {\rho}_{l} {c}_{p,l} & 0 < T \le 99^{\circ}\mathrm{C}\\ {\lambda C}_{H2O}/\Delta {T}_{b} & 99 < T \le 100^{\circ}\mathrm{C}\\ {\rho}_{g}{c}_{p,g} & T>100^\circ{\rm C} \end{array}\right.$$

where ${\rho }_{i}$ and ${c}_{i}$ are density and heat capacity of cardiac tissue before and post phase change, *i* = *l* as liquid phase, and *i* = *g* as gas phase. Water vaporization latent heat, $\lambda$, is 2257 (kJ kg^−1^). Tissue water content, ${C}_{H2O}$, inside cardiac tissue is 75%. Water density at 100 °C is 958 (kg m^−3^) and $\Delta {T}_{b}$ is represented as the temperature difference as assumed by the enthalpy method. Cardiac tissue in liquid phase, $\rho$ is 1060 (kg m^−3^) and ${C}_{p}$ is 3111 (J kg^−1^ K^−1^), while the gas phase, $\rho$ is 370 (kg m^−3^) and ${C}_{p}$ is 2156 (J kg^−1^ K^−1^).

Correspondingly, the result of tissue vaporization would eventuate in desiccation. The desiccation is the dehydration effect that occurred when the cells lose water through the thermally damaged cellular wall. It results in rapid impedance (resistance) increase and then causes lower electrical conductivity. This occurrence will limit thermal volume and lead to less thermal diffusion [34]. The function of electrical conductivity, ${\sigma }^{*},$ and thermal conductivity, ${k}^{*}$, vary by temperature and can be written as temperature dependent functions as given by [9, 10, 13, 22];

17 $${\sigma }^{*}(T)=\left\{\begin{array}{ll}{0.541\mathrm{e}}^{0.015(T-37)}, & 0 < T\le 100^\circ{\rm C}\\ 1.371-0.274\left(T-100\right), & {100} < T \le 10{5}^\circ{\rm C}\\ {1.371\cdot 10}^{-4}, & T >{10}5^\circ{\rm C} \end{array}\right.$$

18 $${k}^{*}(T)=\left\{\begin{array}{ll}0.531+0.0012\left(T-37\right), & 0 < T\le 100^\circ{\rm C} \\ 0.606, &T > 100^\circ{\rm C} \end{array}\right.$$

### Poromechanical deformation analysis

To obtain deformations in the cardiac tissue, a simplified quasi-static poromechanical deformation analysis is used, treating the tissue as a porous medium. The equilibrium equations for solid mechanics, written in a Cartesian coordinate system, are [63]:

19 $$\begin{array}{c}\frac{\partial {\xi }_{xx}}{\partial x}+\frac{\partial {\xi }_{xy}}{\partial y}+\frac{{\partial \xi }_{xz}}{\partial z}=0\\ \frac{\partial {\xi }_{xy}}{\partial x}+\frac{\partial {\xi }_{yy}}{\partial y}+\frac{{\partial \xi }_{yz}}{\partial z}=0\\ \frac{\partial {\xi }_{xz}}{\partial x}+\frac{\partial {\xi }_{yz}}{\partial y}+\frac{\partial {\xi }_{zz}}{\partial z}=0\end{array}$$

The stress–strain relationship (Eq. (20)) and the strain–displacement relationship (Eq. (21)) are as follows [63]:

20 $$\begin{array}{l}{{\gamma }^{m}}_{xx}=\frac{1}{E}\left[{\xi }_{xx}-\nu \left({\xi }_{yy}+{\xi }_{zz}\right)\right]+{\gamma }^{th}\\ {{\gamma }^{m}}_{yy}=\frac{1}{E}\left[{\xi }_{yy}-\nu \left({\xi }_{xx}+{\xi }_{zz}\right)\right]+{\gamma }^{th}\\ \begin{array}{l}{{\gamma }^{m}}_{zz}=\frac{1}{E}\left[{\xi }_{zz}-\nu \left({\xi }_{xx}+{\xi }_{yy}\right)\right]+{\gamma }^{th}\\ {{\gamma }^{m}}_{xy}={\xi }_{xy}\left(1+\nu \right)/E\\ \begin{array}{l}{{\gamma }^{m}}_{xz}={\xi }_{xz}\left(1+\nu \right)/E\\ {{\gamma }^{m}}_{yz}={\xi }_{yz}\left(1+\nu \right)/E\\ {{\gamma }^{m}}_{xx}=\frac{\partial {u}_{x}}{\partial x},{{\gamma }^{m}}_{yy}=\frac{\partial {u}_{y}}{\partial y},{{\gamma }^{m}}_{zz}=\frac{\partial {u}_{z}}{\partial z}\end{array}\end{array}\end{array}$$

21 $$\begin{array}{c}{{\gamma }^{m}}_{xy}=\frac{1}{2}\left(\frac{\partial {u}_{x}}{\partial y}+\frac{\partial {u}_{y}}{\partial z}\right)\\ {{\gamma }^{m}}_{xz}=\frac{1}{2}\left(\frac{\partial {u}_{x}}{\partial z}+\frac{\partial {u}_{z}}{\partial x}\right)\\ {{\gamma }^{m}}_{yz}=\frac{1}{2}\left(\frac{\partial {u}_{y}}{\partial z}+\frac{\partial {u}_{z}}{\partial y}\right)\end{array}$$

where $\xi$ denotes the stress (Pa), ${\gamma }^{m}$ is the mechanical strain, *E* is Young’s modulus (Pa), *v* is the Poisson’s ratio, and *u* is the average displacement (m). The thermal strain, ${\gamma }^{th}$ was calculated as follows:

22 $${\gamma }^{th}={\int }_{{T}_{ref}}^{T}{\beta }_{t}dT$$

where ${T}_{ref}$ =37 $^\circ{\rm C}$ is the reference temperature and ${\beta }_{t}$ is the temperature-dependent thermal expansion coefficient ($^\circ{\rm C}$^−1^) of porous cardiac tissue of temperature at 37 $^\circ{\rm C}$ as indicated in Table 1. The change in porosity after deformation is defined as follows:

23 $$\delta =\varepsilon -{\varepsilon }_{0}$$

24 $$\delta =\frac{{V}_{p}-{V}_{p}^{0}}{d\Omega }$$

where $\delta$ denotes as change in porosity, $\varepsilon$ is porosity, ${V}_{p}$ is volume of pore (m^3^), and $d\Omega$ is total volume of solid matrix (m^3^). Superscript 0 represents as initial value.

#### Boundary condition for poromechanical deformation

As shown in Fig. 4(c), poromechanical deformation is considered only for the cardiac tissue. The boundary condition is set as moving surface for surrounding surfaces of the cardiac tissue domain. Top and bottom surfaces of tissue domain, as well as electrode-tissue interfaces, are fixed. The tissue deforms due to the thermal strain from temperature changes within the tissue domain. There is no contact force in this study. The initial stress and strain are set to zero.

### Numerical computation

**Fig. 5:**
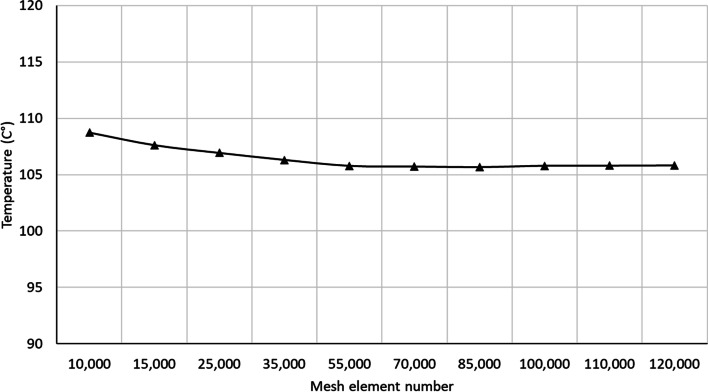
Mesh convergence finite element numerical computation showing sufficient number of elements were used in the computation

The electrical, heat transfer, blood flow and mechanical deformation analysis (Eqns. (2,3,4,5,6,7,8,9,10,11,12,13,14,15,16,17,18,19,20,21,22,23,24) are numerically solved using the finite element method, as implemented in the COMSOL™ Multiphysics software. The 3-D RFCA model is discretized using pyramid elements. A mesh convergence is performed to identify the suitable number of elements required as demonstrated in Fig. 5. This convergence test leads to a grid with approximately 70,000 elements.

## Model validation

**Fig. 6:**
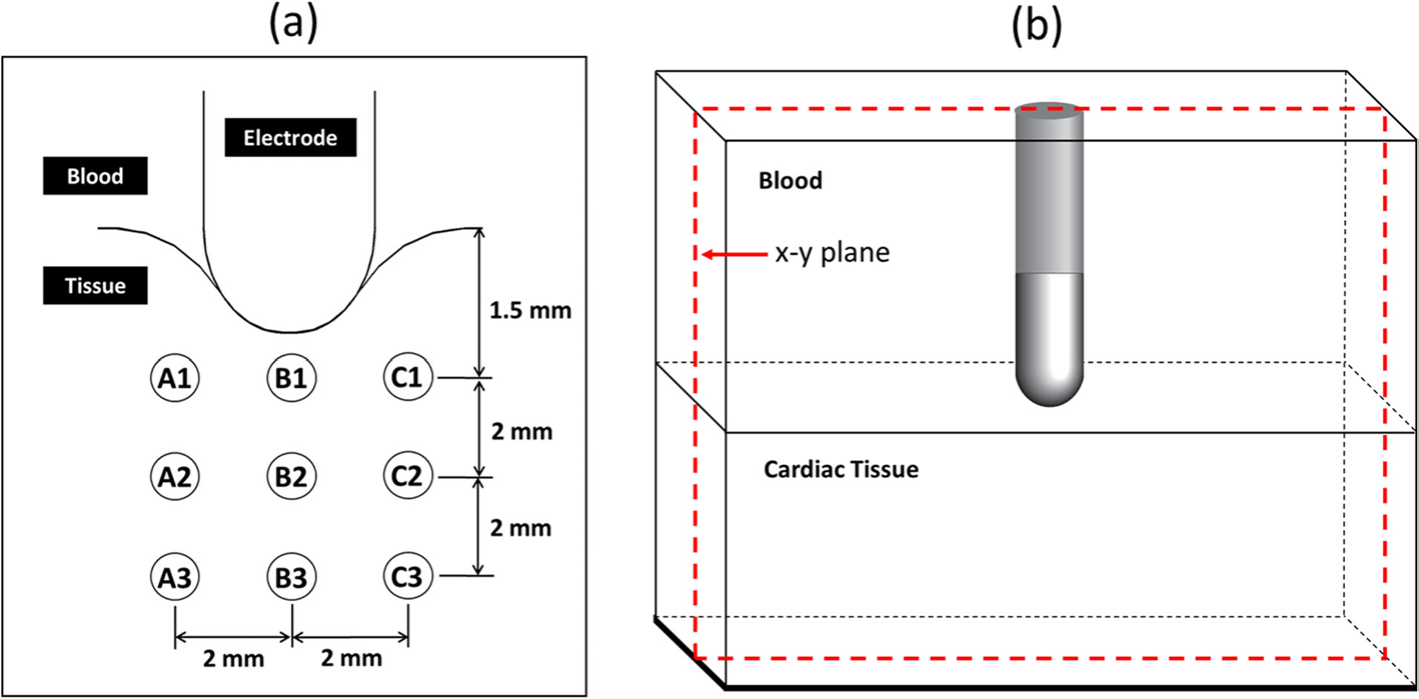
**a** Locations around the electrode within the cross-section, and **b** cross-section through the middle of electrode-tissue domain where computed variables are discussed

**Fig. 7:**
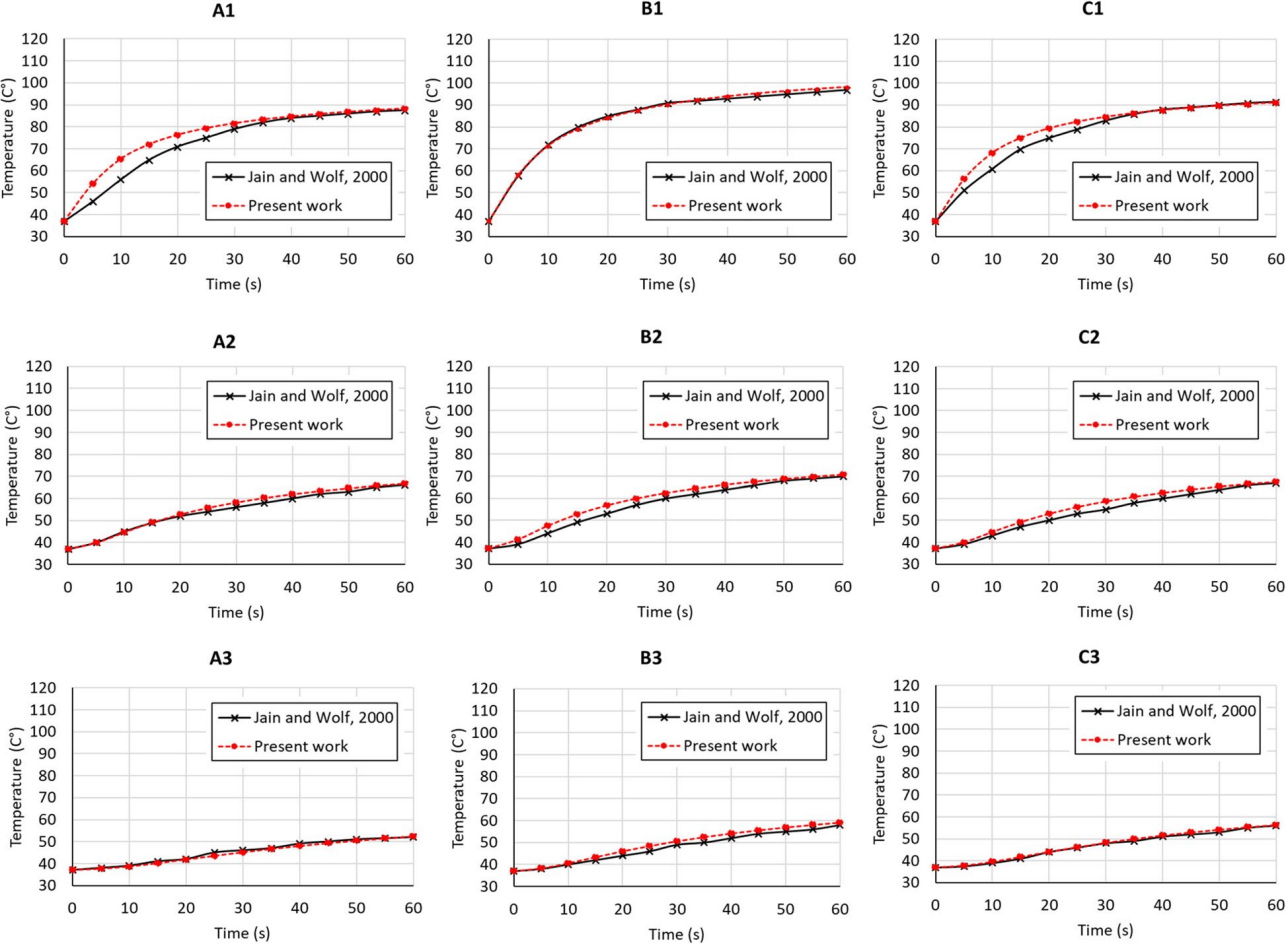
Model validation showing good match between computed transient temperatures and experimental results of Jain and Wolf, 2000 [61]. Locations are those shown in Fig. 6

Figure 7 shows model validation against experimental data [61] for the locations noted in Fig. 6. The model input data were matched to the experimental conditions, with power set at 20 W, inlet velocity in blood domain and porous tissue domain at 3 cm s^−1^ and 0 cm s^−1^, respectively, and the same boundary conditions of electricity, blood flow and heat transfer. Figure 7 shows an excellent comparison between the computational and experimental results, with the largest difference at location A1, likely due to the absence of flow in the experimental non-living tissue. This close comparison validates our model for our conditions that are not substantially different from the experimental work [61].

## Results and discussion

We present here the effects of poromechanical deformation, blood flow, phase change, and inserted electrode depth on higher temperatures as predictors of phase-change associated with steam pop likelihood, using the 3-D RFCA model that treats the tissue as a deformable porous medium. The locations for which data are reported and their positions in the cross-sectional plane are show in Fig. 6a and b, respectively. The power is set at 20 W, with the inlet velocities in blood and tissue layers set as 3 cm s^−1^ and 2 m$\mu$ s^−1^, respectively. The external load is set at 0 N.

### Poromechanical deformation reduces flow velocities and increases temperature

**Fig. 8:**
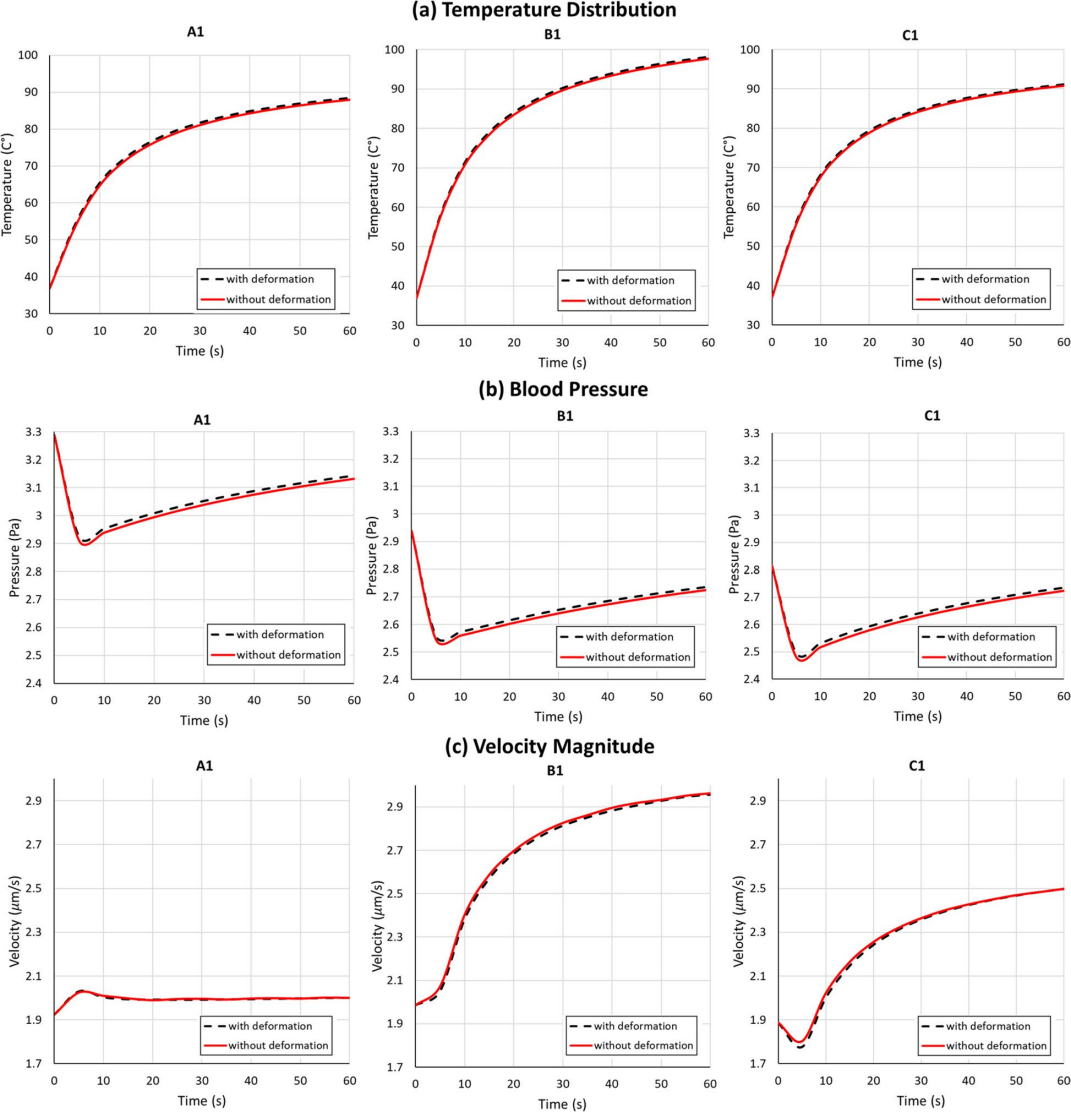
Effect of thermally driven poromechanical deformation on computed temperature, pressure, and velocities, showing a small effect

**Fig. 9:**
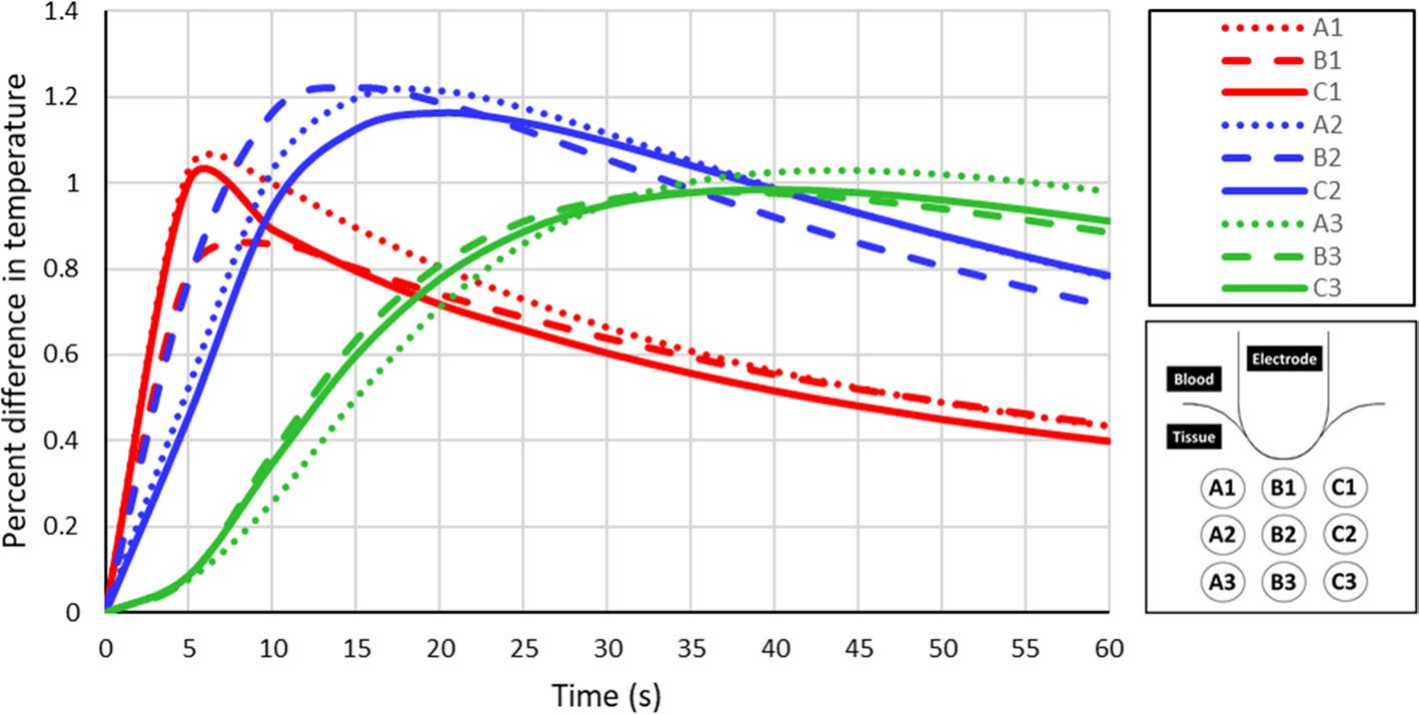
Impact of deformation on computed temperatures at the locations shown in Fig. 6. Percent difference is calculated as $\frac{{\mathrm{T}}_{\mathrm{deform}}-{\mathrm{T}}_{\mathrm{undeform}}}{{\mathrm{T}}_{\mathrm{undeform}}}(^\circ \mathrm{C})\times 100\mathrm{\%}$

The significance of poromechanical deformation in the tissue becomes evident in its effects on blood flow and temperature distribution. Figure 8 shows that poromechanical deformation in the tissue raises its temperature. Tissue expands at a higher temperature, increasing its porosity and permeability, reducing the flow velocities and the resulting natural convection heat transfer. Figure 9 shows that the influence of thermomechanical expansion of the tissue on its temperature is initially the highest at locations closest to the electrode (A1, B1, and C1) and, as heat diffuses with time, this influence moves to the more distant locations (A3, B3, and C3).

**Fig. 10:**
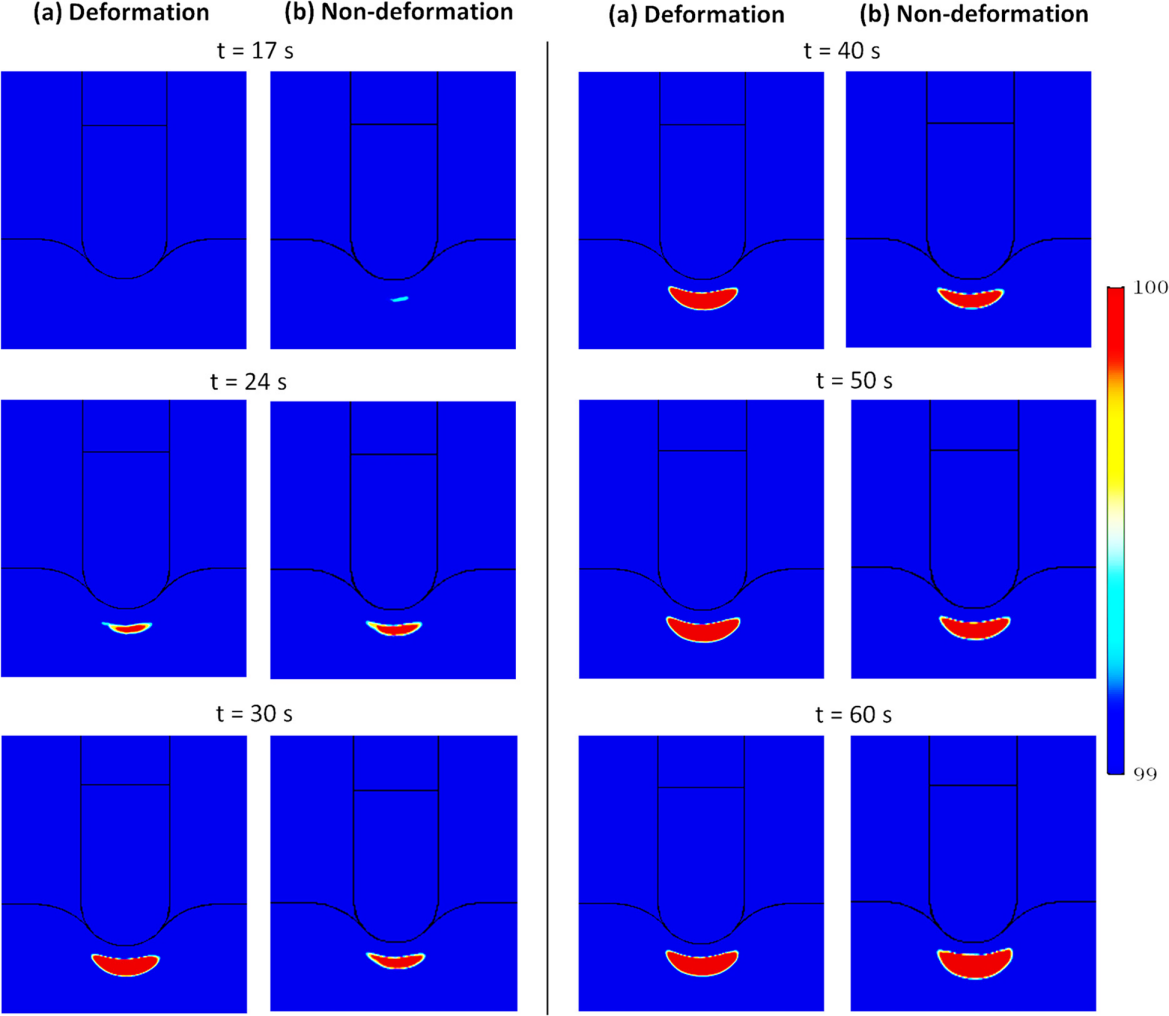
Effect of inclusion of poromechanical deformation on the expected region for steam pop (temperatures 99 °C or higher) in the cardiac tissue at different times during procedure

When steam pop is expected to occur over time and the region over which it is expected to occur (Fig. 10), are a complex function of the thermomechanical expansion of the tissue (that decreases flow with temperature) and thermally driven natural convection (that increases flow with temperature gradient). As shown in Fig. 10, with deformation, steam pop is expected to occur later but the region over which it is expected to occur is larger, for the most part, but not always.

Property changes due to phase change are incorporated by making them function of temperature (Eqs. 16, 17 and 18) covering the phase transition range. This includes density, heat capacity, electrical conductivity, and thermal conductivity. The poromechanical model is affected by the temperatures that are in turn dependent on the extent of liquid–gas transition (Eq. 16) and all other property changes with temperature. The expansion or shrinkage of pores, in turn, can affect the pore pressure, as well as thermal conduction and thermal convection.

When comparing the temperature distribution among different models, it was observed that a 3D model without symmetry produced different heat spots compared to the corresponding 2D model with axial symmetry [14]. Additionally, incorporating porous media flow led to further asymmetry in the temperature distribution due to thermal convection, in contrast to the bioheat model [9]. Consideration of natural convection and poromechanical deformation also resulted in variations in the hot spots, attributed to changes in blood flow direction and the ability of blood to flow through pores, which differed from models that did not consider these effects [22].

### Decrease in tissue permeability reduces flow, increasing temperature and the likelihood of steam pop

The tissue properties of porosity and permeability affect the flow and temperature computations and thus it is important to know their effects. Changes in these transport properties of cardiac tissue can naturally occur from, for example, changes in blood vessel size with locations. A range of porosity and permeability values for tissues were estimated from the reported blood vessel sizes, using Eqns. 10 and 11, and used to calculate their effects on temperature and velocities.

Higher porosity allows for greater deformability of the tissue and facilitates better fluid flow, ensuring adequate blood supply to the tissue. It also enhances convective heat transfer, resulting in efficient cooling and a more uniform temperature distribution. Conversely, lower porosity or permeability can impede blood flow, leading to insufficient tissue perfusion. It also reduces convective heat transfer, potentially causing localized temperature elevations, thus steam pop is expected to happen earlier due to the higher temperatures reached for lower porosity and permeability.

**Fig. 11:**
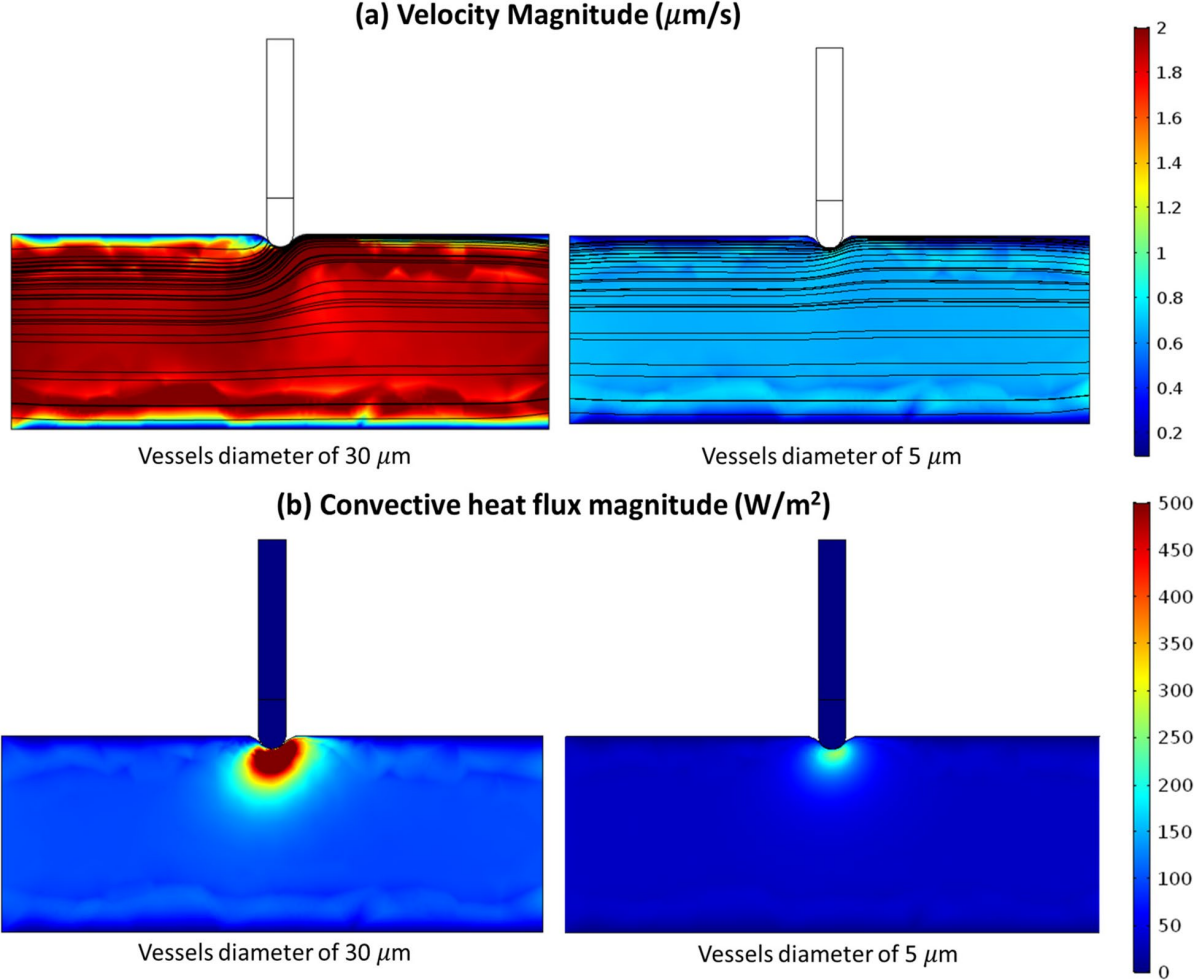
Blood velocity magnitude **a** and convective heat flux **b** in the cardiac tissue for two different blood vessel diameters (representing two different porosities) after 60 s of heating. Poromechanical deformation is included here

**Fig. 12:**
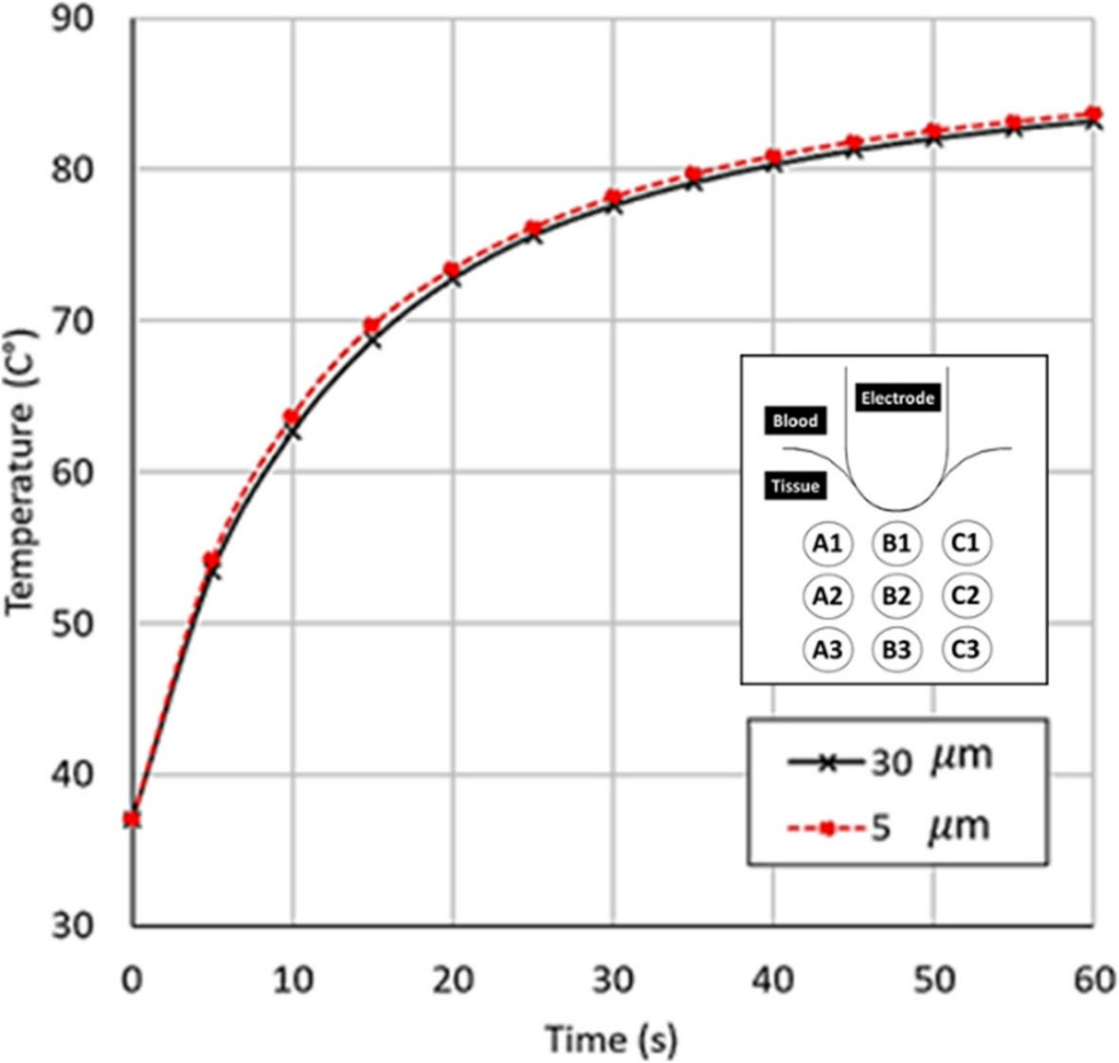
Transient temperatures at location B1 (Fig. 6a) of two different blood vessel diameters (representing two different porosities). Poromechanical deformation is included here

**Fig. 13:**
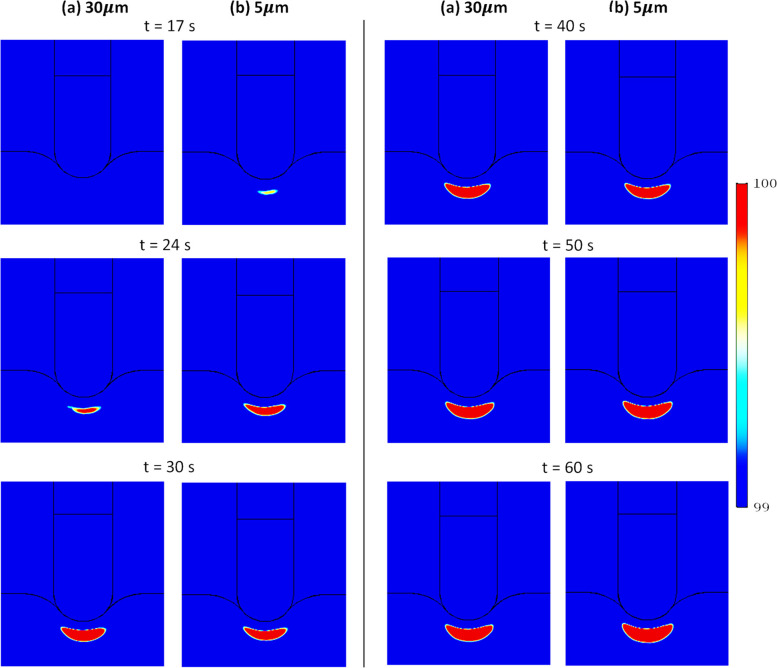
Effect of porosity (as estimated from blood vessel diameter) on the expected region for steam pop (temperatures 99 °C or higher) in the cardiac tissue at different times during procedure, for two vessel diameters: **a** 30 m$\mu$ and **b** 5 m$\mu$. Poromechanical deformation is included here

Figure 11a shows that lower permeability (corresponding to smaller blood vessels) makes it harder for fluid to flow (i.e., lower velocity in the right figure). This lower velocity with reduced convective heat flux (Fig. 11b) leads to a slightly higher temperature (Fig. 12) since the cooling effect of convection is reduced. This ultimately affects steam pop, as shown in Fig. 13. For lower permeability, steam pop is expected to occur sooner (due to higher temperatures) and, over time, is mostly over a larger area (due to reduced thermal convection).

### Deeper electrode insertion raises temperature due to increased contact surface

**Fig. 14:**
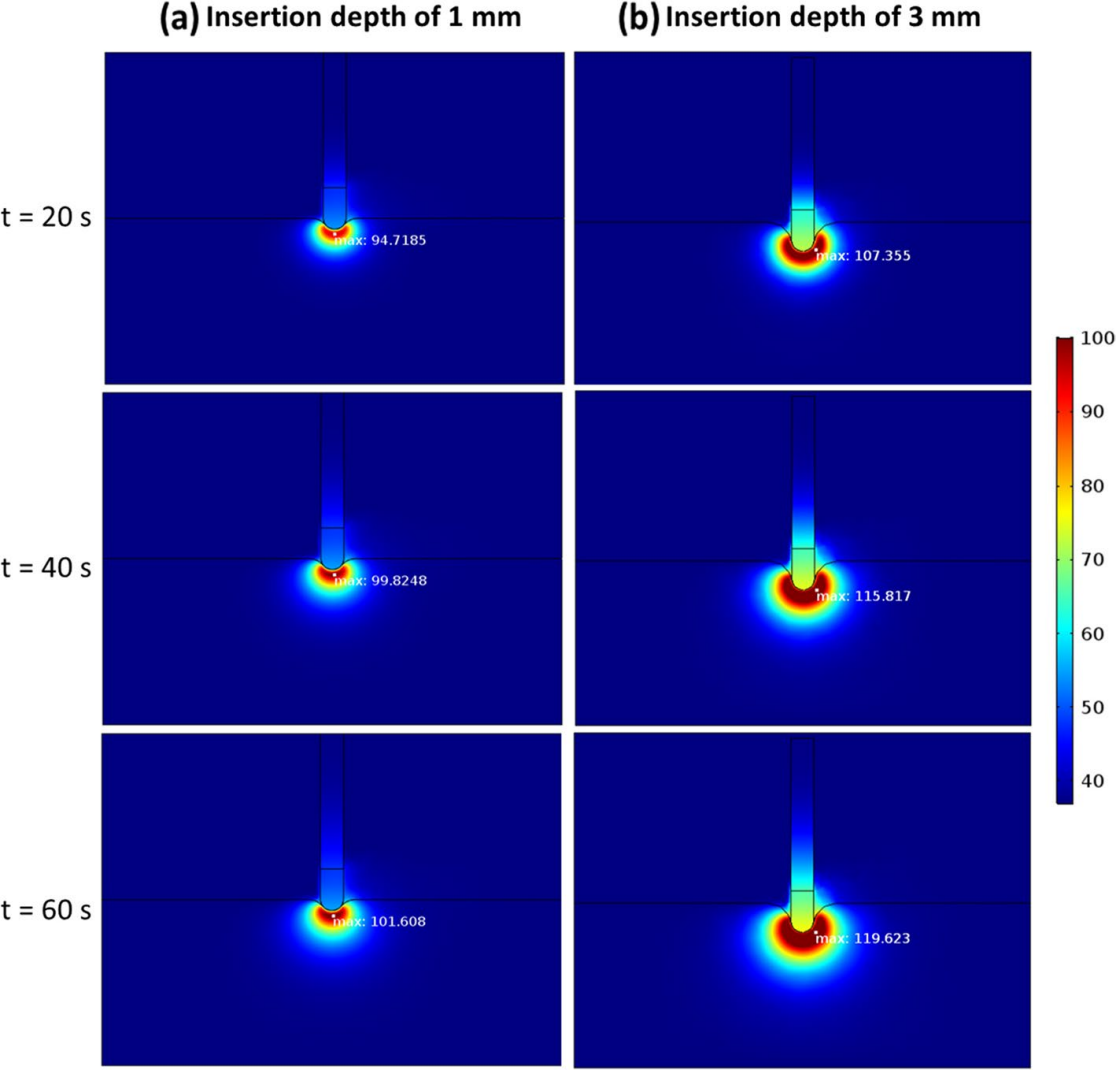
Effect of electrode insertion depth (1 mm and 3 mm) on temperatures in the cardiac tissue at different times during procedure. Poromechanical deformation is included here

Two electrode insertion depths of 1 and 3 mm were chosen to represent the effect of tissue shrinkage and expansion during the systole and diastole phases of the cardiac cycle. Corresponding geometries were computed using Eq. 1. As shown in Fig. 14, deeper insertions lead to higher temperatures and larger hot spots within the tissue. The increased contact between the electrode and the tissue at deeper insertion allows more electrical current to flow, generating more heat. The higher temperatures from increased heat generation can lead to greater pore expansion in the tissue, reducing velocities (convection) and thus making it even hotter. Additionally, the increased energy deposition from the greater current flow raises the likelihood of steam pops occurring more frequently.

**Fig. 15:**
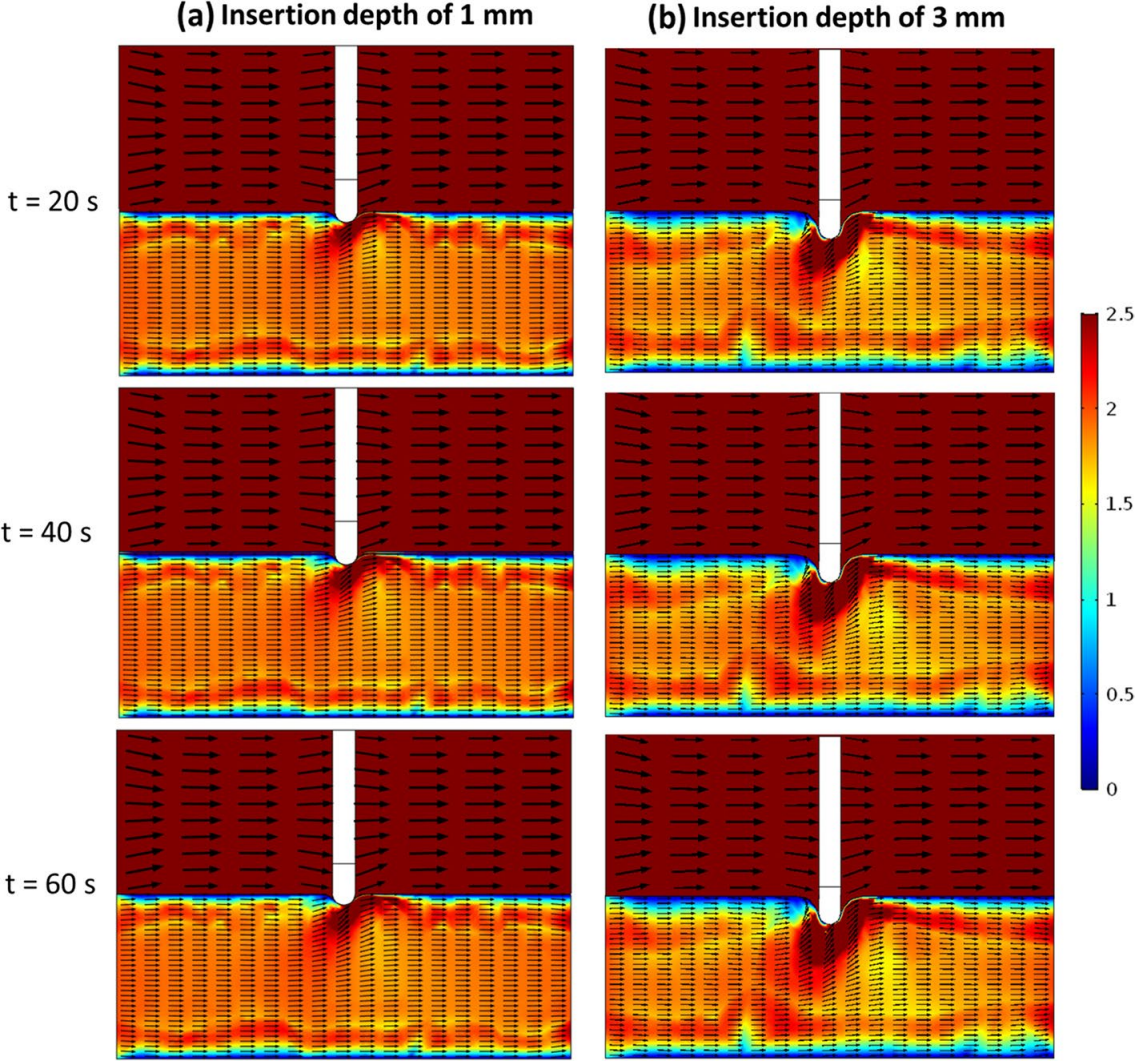
Effect of electrode insertion depth (1 mm and 3 mm) on flow magnitude and direction in the cardiac tissue and blood domains at different times during procedure. Poromechanical deformation is included here

Both natural and forced convection of blood change the hot spot. As shown in Fig. 15, forced convection makes the hot spot asymmetric, with more of the hot region on the downstream side, true for both insertion depths. The natural convection effect is stronger for the higher temperatures (more buoyant force) resulting from deeper insertion, leading to larger velocity magnitudes and increased upward flow. Although natural convection effects are stronger for the deeper insertion, heat generation from current flow still dominates in raising the temperature.

The higher temperatures resulting from deeper insertion increases the likelihood of steam pop occurrence. Since the rate of temperature rise is also faster (more energy deposition due to higher current flow), steam pops will likely be more frequent.

## Conclusions

A comprehensive understanding of RFCA was achieved through a coupled electrical, thermal, poromechanical deformation, and fluid transport model of the process. Blood flow from normal perfusion and buoyancy introduced by the temperature gradients of the RFCA reduces the temperatures but also heats an expanded area. The poromechanical expansion due to temperature gradients leads to increased porosity and decreased blood velocity, thereby increasing tissue temperature. Tissue properties of porosity and permeability influence temperatures, with lower porosity increasing the temperatures slightly, due to lower velocities. Deeper electrode insertion, representative of tissue expansion/contraction during systolic/diastolic phases, raises temperature due to increased contact surface area between the electrode and tissue.

The multiphysics model utilized in this study provides insights into temperature distributions within the tissue, enabling an understanding of the impact of thermo-poromechanical deformation of the tissue by considering parameters such as porosity, permeability, and pore thermal expansion. Notably, the results demonstrate that a 5% increase in porosity leads to a significant 10% increase in maximum tissue temperature. These findings are supported by previous research, e.g.: Deformation affects the flow in porous media and the mass of blood flow in small arteries and veins within myocardial tissue [64]. This relationship between deformation and blood flow has implications for temperature regulation through thermal convection. Additionally, experimental evidence of thermal strain in arteries [65] aligns with our understanding of pore expansion due to heat and its effects on blood flow dynamics.

The significance of poromechanical deformation in this work distinguishes it from previous modeling studies. While previous approaches mainly focused on blood flow and temperature distribution within the tissue, this study takes into account the impact of poromechanical deformation on blood flow and temperature distribution. By incorporating deformation, the model achieves a more comprehensive understanding of the intricate interplay between tissue volume, porosity, permeability, and thermal expansion, which impact blood flow and temperature distribution. Consequently, this approach enables a more accurate representation of the real tissue structure and yields results that are closer to reality compared to previous models that neglected pore deformation. Additionally, this study investigates the effects of different electrode insertion depths on heat transfer and blood flow through the tissue, which has not been extensively studied before.

The limitation of this study is that it predicts only indirectly the steam pop likelihood, by the presence of higher temperatures. The study may not fully capture the steam pop dynamics that could come out from the prediction of gas pressure by considering multiphase (gas, liquid) transport, distributed evaporation, and gas pressure-driven deformation [29] in addition to thermo-poromechanics and voltage modeling. Such a model would be another level more complex, the need for which can only be justified by the results of a less complex model like this one. As was shown, this model validated against the experimental data reasonably well. Nevertheless, it could be worthwhile to consider developing such a model that could improve the prediction, leading to potentially better management of steam pops during a procedure, ultimately enhancing patient safety and treatment outcomes.

## Nomenclature

${C}_{H2O}$ Tissue water content wet basis (fraction)

${C}_{p}$ specific heat capacity (J kg^−1^ K^−1^)

$\left|\mathrm{\rm E}\right|$ magnitude of the electric field (V m^−1^)

$E$ Young’s modulus (Pa)

*K *permeability (m^2^)

*Q *external heat source (W m^-3^)

*R *radius of the electrode tip (mm)

*S*_*v*_ specific surface area (m^-1^)

*T *temperature ($$^\circ{\rm C}$$)

*U *velocity of blood (m s^-1^)

${V}_{p}$ volume of pore (m^3^).

*a *contact radius (mm)

*d* capillary diameter ($$\upmu$$ m)

$$g$$ gravity (m s.^−2^)

$$k$$ thermal conductivity (W m^−1^ K.^−1^)

*p *pressure (Pa)

*r *radius of pores or vessels within tissue ($\upmu$ m)

*t *time (s)

*u *displacement (m)

**Greek letters**

${\beta }_{t}$ temperature-dependent thermal expansion coefficient ($^\circ{\rm C}$^−1^)

$\delta$ change in porosity (-)

$$\varepsilon$$ tissue porosity (-)

$${\varepsilon }_{r}$$ relative permittivity (-)

$\kappa$ volume fraction of pores (-)

$\lambda$ latent heat of vaporization of water (kJ kg^−1^)

$\mu$ kinematic viscosity of blood (kg m^−1^ s^−1^)

*v *Poisson’s ratio (-)

$$\xi$$ stress (Pa)

$$\rho$$ density (kg m^−3^)

$$\sigma$$ electrical conductivity (S m^−1^)

$$\tau$$ tortuosity (-)

$$\gamma$$ strain (-)

$\varphi$ electric potential or voltage (V)

$d\Omega$ total volume of solid matrix (m^3^)

${\omega }_{b}$ blood perfusion rate (s^−1^)

${\omega }_{max}$ maximum vertical displacement (mm)

**Subscripts**

0 initial value

RF radiofrequency

met metabolic

eff effective value

b blood phase

l liquid phase

g gas phase

ref reference value

**Superscript**

0 initial value

m mechanical

th thermal

## Data Availability

The authors declare that the data supporting the findings of this study are available within the paper.
